# Generalized Additive Modeling of Ecological Data With mgcv: New Adequacy Assessment Tools

**DOI:** 10.1002/ece3.72825

**Published:** 2026-01-09

**Authors:** Julien Mainguy, Rachel McInerney, Russell B. Millar, Eliane Valiquette, Martin Bélanger, Rafael de Andrade Moral

**Affiliations:** ^1^ Direction principale de l'expertise sur la faune aquatique Ministère de l'Environnement, de la Lutte contre les changements climatiques, de la Faune et des Parcs Québec Québec Canada; ^2^ Department of Mathematics and Statistics Maynooth University Maynooth Ireland; ^3^ Department of Statistics University of Auckland Auckland New Zealand; ^4^ Direction de la gestion de la faune de l'Abitibi‐Témiscamingue Ministère de l'Environnement, de la Lutte contre les changements climatiques, de la Faune et des Parcs Rouyn‐Noranda Québec Canada

**Keywords:** adjusted deviance explained, deviance residuals, half‐normal plot with a simulated envelope, mgcViz score, under‐ and overfitting

## Abstract

Generalized additive models (GAMs) are a semi‐parametric extension of generalized linear models (GLMs) that allow incorporating different forms of nonlinearities commonly encountered in ecological relationships, thus frequently offering a better statistical description than GLMs in such cases. Due to the use of smooth functions, however, validating that the observed data represent a plausible realization of a fitted GAM according to the underlying distributional assumptions being used is less straightforward than with GLMs. Moreover, if the number of basis dimensions used in smooth terms to control the degree of flexibility is set too large, overfitting can arise despite in‐built penalization procedures aimed at preventing excessive wiggliness. Here, we present how GAMs fitted with the mgcv package in R can be assessed for their adequacy based on half‐normal plots with a simulated envelope using newly‐available helper functions for the hnp package. A proposed metric relying on the mgcViz package is also presented to help detect both under‐ and overfitting relative to a predictor of interest from a realized coverage perspective. Three fisheries‐related examples analyzing continuous data, counts, and discrete proportions are then presented to illustrate the usefulness of these approaches in providing more statistical context for the interpretation of nonlinear ecological relationships.

## Graphical abstract

Anadromous Nunavik Arctic charr ( *Salvelinus alpinus* ) during the upstream migration in the Aipparusik (Bérard) River in September 2017, Tasiujaq, Québec, Canada. Sampled female Arctic charr from this population and two other populations in Nunavik were jointly analyzed to estimate their length‐at‐maturity. This was achieved with the analysis of discrete proportions (i.e., number of females exhibiting developing gonads within 50‐mm fork length bins) using the gam() function of the mgcv package. The adequacy assessment tools presented in this study were applied to this real‐case example (photo: Pascal Ouellet, MELCCFP).

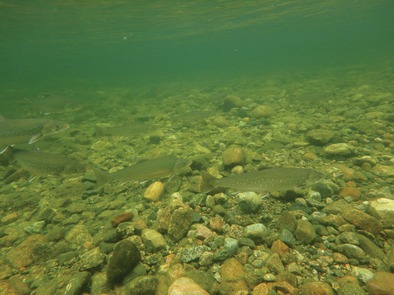

## 1 Introduction

The analysis of diverse types of ecological data is frequently performed with generalized linear models (GLMs) due to their renowned analytical flexibility and associated advantage of producing directly interpretable coefficients at the linear predictor scale (Bolker et al. 2009; Zuur et al. 2009). However, GLMs are frequently limited in their ability to statistically describe nonlinear relationships that can differ from the specific shapes imposed by the inclusion of polynomial terms (e.g., quadratic) but are nonetheless commonly encountered in ecology (Heit et al. 2024). In such cases, a semi‐parametric GLM extension, the generalized additive model (GAM), often provides a better alternative to statistically describe these more complex curvilinear relationships (e.g., Aubry et al. 2009). This is mainly attributable to the ability of a GAM to automatically incorporate different forms of nonlinearities through the application of smooth functions (Hastie and Tibshirani 1986; Wood 2017). As a result, GAMs have become increasingly employed alongside GLMs in ecology (van der Burg et al. 2010; Nelsen et al. 2023; Rodriguez‐Cordero et al. 2024; Mainguy, Paradis, and Moral 2025), but contrary to GLMs, their parameter coefficients are not directly interpretable such that visualization of the fitted values is required for their interpretation. Both GLM and GAM fits, however, need to sufficiently respect their underlying distributional assumptions to reach model adequacy and thus yield reliable statistical inferences (Guisan et al. 2002).

Due to their use of smooth terms, the adequacy assessment of GAMs is generally less straightforward than for GLMs. Examination of the distribution of different types of residuals with diagnostic plots, as normally used for GLMs (Zuur et al. 2009; Millar 2011), can however also be achieved with GAMs. The Quantile‐Quantile (QQ) plot (Augustin et al. 2012) stands out for this as it allows us to determine whether the residuals of a considered model are distributed according to expected theoretical quantiles. A worm plot (van Buuren and Fredriks 2001), which corresponds to a detrended QQ plot, is also of interest as an alternative display. Despite their utility, such visual checks have an inherent subjectivity that can sometimes lead to contradicting interpretations about whether a model is sufficiently adequate, unless an obvious problematic residual pattern is detected, such that more objective complementary assessments should ideally also be performed.

Adequacy assessment approaches that evaluate how well a given type of residual “behaves” are often derived from diagnostic plots from which different quantitative or statistical tests can be conducted (e.g., Hartig 2024). Such additional objective evaluations of adequacy can ultimately allow to more reliably select a “best” model among a set of a priori identified adequate candidate ones, such that the one retained is more likely to be of practical use (Mac Nally et al. 2018). If all the considered candidate models fail at some (if not all) adequacy checks, then this may at least provide the opportunity to identify the nature of the potential shortcomings, such as using an incorrect distributional assumption (Moral et al. 2017). As an example, modeling overdispersed counts when solely relying on the Poisson distribution would lead to such an outcome (Richards 2008). Even if the data‐generating process is not adequately represented by the preferred model, it can nonetheless remain useful to some extent when interpreted as such (Odenbaugh 2005).

As GAMs are also predominantly data‐driven from their use of smooth terms (Guisan et al. 2002), this makes them prone to overfitting by sometimes potentially capturing more noise from the data than the true underlying signal (Pedersen et al. 2019). Because overfitting is often associated with excessive wiggliness for a fitted GAM, which may occur despite in‐built penalization procedures (e.g., Wood 2017), the degree of added nonlinearities for a main predictor of interest may need to be checked. For instance, the subspace of functions employed in a GAM when fitted with the mgcv package (Wood 2017) is sometimes voluntarily constrained for a main smooth term being considered by manually reducing the number of basis dimensions (*k*) that it can use to minimize wiggliness (e.g., Dehnhard et al. 2020; Rodriguez‐Cordero et al. 2024; Kaplan et al. 2025). Under this approach, however, underfitting may occur instead, such that developing reliable procedures to objectively assist in the detection of both under‐ and overfitting is desirable to aid in identifying such situations for a given predictor.

Here, we describe two approaches that can be applied for the adequacy assessment of GAMs fitted with mgcv. More specifically, using three fisheries‐related examples dealing with either continuous data, counts, or discrete proportions from the Ministère de l'Environnement, de la Lutte contre les changements climatiques, de la Faune et des Parcs (MELCCFP, Québec, Canada) analyzed with mixed‐effects GAMs (GAMMs), the application of half‐normal plots with a simulated envelope made possible due to new helper functions for the hnp package (Moral et al. 2017) is first demonstrated. Then, a novel metric relying on the mgcViz package (Fasiolo et al. 2020) is described to determine, from a realized coverage perspective, the possible occurrence of under‐ and overfitting relative to an included smooth term or parametric component of interest.

The open‐source R statistical software (R Core Team 2025) was employed due to its increasing popularity for the analysis of ecological data (Lai et al. 2019), with mgcv incontestably being one of the most preferred (Gao et al. 2025). The analyses presented in this study were thus limited to mgcv as it also benefits from useful diagnostic tools, such as those implemented in the DHARMa (Hartig 2024) and itsadug (van Rij et al. 2022) packages. To allow reproducibility of the results presented in this study, MELCCFP datasets and R code have been made publicly available at https://github.com/julienmainguy/GAM‐adequacy.

## 2 Model Adequacy

Diagnostic tools for adequacy assessment predominantly rely on inspecting residuals, the simplest of which are the response (i.e., raw) residuals. However, alternative types of residuals offer better properties when the response variable is non‐Gaussian, such as the deviance and Pearson residuals (Millar 2011), although other types may also be used for the same purposes, such as the quantile residuals obtained from model‐based simulations in DHARMa. Regarding the deviance residuals, they are generally preferred over the Pearson residuals for diagnostic purposes (Zuur et al. 2009), and accordingly, they correspond to the default type within mgcv. Beside the examination of diagnostic plots relying on the distribution of the deviance residuals, their useful properties can also be exploited in a more quantitative manner.

### 2.1 Adequacy Assessment

#### 2.1.1 The hnp Package: Empirically Based Simulations for Adequacy Assessment

Half‐normal plots with a simulated envelope (Atkinson 1985) have been used as an intuitive diagnostic tool to determine in most instances whether the distributional assumptions of linear models and GLMs have been sufficiently respected (e.g., Antoniazzi et al. 2019; Mainguy and Moral 2021). The hnp package offers an implementation of this approach by performing many simulations from the fitted model to establish the limits of an envelope in which most of the deviance (or Pearson) residuals should be enclosed if the distributional assumptions upon which the model was built are sufficiently respected (Moral et al. 2017). The envelope limits reflect as a default setting the 2.5th and 97.5th percentiles of the resulting simulated distribution of expected residuals, such that a well‐fitting model should have approximately 95% of its residuals enclosed.

To provide a quantification of adequacy, hnp calculates the percentage of residuals that are found outside the envelope limits. Since these were determined by simulations, using the mean percentage from a series of 10 diagnostics or more is generally recommended and, as a guide, should be < 5% for an adequate fit and between 5% and 10% for an acceptable fit, as suggested by Mainguy and Moral (2021). A single half‐normal diagnostic plot exhibiting a large percentage of residuals found outside the envelope (e.g., 47%) is already indicative of an inadequate model, but a value of 11% may be followed by one of 4% for a second hnp iteration, such that performing many diagnostics to obtain a central tendency is preferable. This is especially true with small sample sizes (*n* < 30), given that each residual found outside the envelope will impact more importantly the resulting mean percentage, such that a visual examination is also recommended in such cases (Mainguy and Moral 2021).

The modal percentage may however represent a more reliable descriptive statistic to avoid any possible leverage effect on the mean of a single low or high percentage value that could have arisen from a low number of diagnostic iterations. The recommendation of using 10 hnp diagnostics, each based on 99 simulations as a default setting, is considered to constitute a reasonable compromise since this analytical process can be computationally intensive, especially for highly parameterized GAMMs. The helper functions now allow the assessment of GAMs and GAMMs fitted with commonly employed distribution families in ecology, namely the Gaussian, binomial, Poisson, type‐II negative binomial (NB2), and gamma with log link (Bolker et al. 2009; Zuur et al. 2009), and may eventually be integrated in a future release of hnp.

#### 2.1.2 The mgcViz Score: Detection of Under‐ and Overfitting

Model‐based simulations performed with mgcViz provide a diagnostic plot for GAMs fitted in mgcv only, allowing to visually assess how the means of binned deviance residuals are distributed with respect to a simulated 80% confidence interval as a default setting. As such, for a correctly specified GAM, approximately four means out of five are expected to be found within the 80% CI limits. The proposed mgcViz score is simply obtained by performing 100 diagnostic iterations, each based on 100 model‐based simulations, to quantify the realized coverage mean (%) of a candidate GAM. Under this rationale, an mgcViz score > 80% suggests overfitting and more prominently so as this score moves closer to 100%, whereas the opposite pattern suggests underfitting. A 95% “uncertainty” interval for the proposed mgcViz score can also be estimated from the 2.5th and 97.5th percentiles of the resulting distribution, which should ideally include the targeted 80% value.

### 2.2 Application to Ecological Data

#### 2.2.1 General Modeling Approach

All statistical analyses were performed using the mgcv default settings for smooth terms, that is, relying on thin‐plate regression splines with *k* = 10 (Wood 2017), except in one instance for which an adaptive smooth relying on penalized splines (still using *k* = 10) was used instead to better accommodate a required biological reality (see Section 2.2.2). Given that all examples included fish collected from different sampling locations, random effects were used in all instances to account for the structure of dependency in the data (Millar and Anderson 2004). All candidate GAMMs were initially fitted using maximum likelihood (ML) estimation to enable more reliable comparisons since they shared an identical random structure but differed for their fixed effects (Zuur et al. 2009). When a GAMM included many smooth terms fitted as additive effects (see Section 2.2.3), the “double‐penalty” approach of Marra and Wood (2011) was first applied using the argument select = TRUE to further increase the penalization imposed on the smoothing functions as part of a first analytical step, as similarly done in other ecological studies (e.g., Becker et al. 2020). This allowed to identify smooth terms that may have little to no statistical association with the response variable if their associated effective degrees of freedom (edf) shrank toward zero. Briefly, the edf of a smooth term capture the degree of added nonlinearities and progressively moves away from unity when complexity increases (i.e., edf > 1), whereas edf = 1 indicates linearity instead (Zuur et al. 2009). Once unimportant smooth terms were removed, the reduced GAMM was refitted without the initial procedure and any remnant smooth terms that would exhibit edf = 1 were refitted as parametric components instead to ultimately yield a final model under REML estimation for statistical inferences.

Although the step of model selection is not the main focus of this study, efforts were nonetheless made to identify the best candidate GAMM using two complementary approaches: (i) a *χ* ^2^‐test implemented in the compareML() function of itsadug, and (ii) an information‐theoretic approach using the sample‐adjusted Bayesian Information Criterion (BIC_c_; McQuarrie 1999) with the MuMIn package (Bartoń 2025). This specific information criterion was used to favor simplicity while also correcting for small sample size when needed. For (i), the respective complexities of two candidate models being compared are captured by their Estimated degrees of freedom (Edf) which account for the related parametric component degrees of freedom (df), smooth term edf, and penalty null‐space dimensions of each smooth object (van Rij et al. 2022). The *χ* ^2^‐test is used to determine whether the more‐complex model offers a sufficient statistically improved fit compared to that of the less‐complex one based on their respective ML scores and Edf differences (van Rij et al. 2022), as sometimes used in ecological studies (e.g., Dehnhard et al. 2020; Mainguy, Paradis, and Moral 2025). When models' Edf are identical, no *χ* ^2^‐test is performed and the one with lowest ML score is then identified as the one preferred, noting however that a ML score difference < 5 may be considered mostly inconsequential (van Rij et al. 2022).

Once a top‐ranking model was identified, it was refitted under restricted ML (REML) estimation for prediction purposes to minimize the bias in the predicted variance of the random effects since REML estimation accounts for the df lost in fitting the fixed effects (Zuur et al. 2009) and is also generally preferred with mgcv (Pedersen et al. 2019). The in‐sample predictive performance of the top‐ranking model was also estimated with the deviance explained (*D* ^2^), as directly provided together with the adjusted *R* ^2^ (*R* ^2^_adj_) by the gam() function. An adjustment to *D* ^2^ was also applied (*D* ^2^_adj_) following Guisan and Zimmermann (2000).

#### 2.2.2 Length‐at‐Age Relationship in Female Lake Trout

This first example involves the analysis of a continuous response variable by statistically describing the simple curvilinear length‐at‐age relationship of female lake trout ( *Salvelinus namaycush* ; *n* = 153) that were sampled in three different lakes (Mainguy and Beaupré 2019). All sampled female lake trout were measured for their total length (*TL*; ±1 mm) and had their *age* in years determined from one otolith sagittae. Modeling length‐at‐age relationships in fish is predominantly achieved using growth equations fitted in nonlinear least‐squares regressions, such as the commonly used von Bertalanffy growth model (Katsanevakis and Maravelias 2008). Katsanevakis and Maravelias (2008) however suggested that more flexible, nonparametric approaches such as GAMs could potentially better describe the length‐at‐age relationship of fish species compared to conventional growth models that rely on theoretical concepts, such as an asymptotic length‐at‐infinity, but have rarely been applied (e.g., Rigg et al. 2025).

Following this suggestion, the growth of the sampled female lake trout was first analyzed with a GAMM fitted with a Gaussian distribution and ML estimation, as in a mixed‐effects von Bertalanffy growth model, but this led to unrealistic negative (nonmonotonic) growth due to detectable wiggling, such that the use of thin‐plate regression splines was judged inappropriate. To correct for this, penalized splines were instead used within an adaptive smooth (Wood 2017) to impose a greater penalty against wiggliness attributable to the presence of sparsely distributed lengths at older ages, again using the Gaussian distribution. However, because length variation commonly increases with fish age (Rigg et al. 2025), a gamma distribution (log link) was also considered to potentially better capture this biological reality.

#### 2.2.3 Temporal Trend in the CPUE of Small St. Lawrence River Walleyes

In this second example analyzing counts, a temporal trend is modeled to describe how an abundance index (catch‐per‐unit‐effort; CPUE) of small‐sized St. Lawrence River walleyes ( *Sander vitreus* ) with *TL* < 381 mm and aged ≥ 1 year old has varied over a 21‐year timespan (2001–2021) based on periodic gillnet surveys conducted in eight interconnected areas (see Mainguy, Paradis, and Moral 2025 for details). The number (*N*) of small walleyes captured per station showed considerable variation with an overall mean of 2.1 and associated variance of 10.7 (*n* = 1744 year‐stations), indicating that these counts were likely overdispersed (variance > mean) and also possibly zero‐inflated given that empty gillnets were retrieved 38.5% of the time. As overdispersion and zero‐inflation, which sometimes co‐occur, can impact statistical inferences (Richards 2008; Campbell 2021), they were also assessed regarding model adequacy.

As a first step, a fully‐parameterized GAMM fitted with the Poisson distribution and a log link was used to model how *N* varied according to *year* (continuous) fitted as a smooth term, while controlling for other sources of variation using three environmental (i.e., water temperature, conductivity, and turbidity) and three operational covariates (i.e., sampling date, and gillnet fishing depth and soak time), all also fitted as (continuous) additive smooth terms, along with *area* fitted as a random effect (see Mainguy, Paradis, and Moral 2025 for details). Overdispersion and zero‐inflation tests implemented in DHARMa were then performed and indicated that counts in the fully‐parameterized Poisson GAMM were overdispersed with an estimated dispersion parameter ($\hat{\phi }$) of 3.56 (*p* < 0.001) and also zero‐inflated (*p* < 0.001) by predicting only 65% of the observed zeros. On the sole basis of the first overdispersion test, this Poisson GAMM was categorized as inadequate and thus discarded given that the assumption of equidispersion that it relied on was not respected. The same conclusion was unambiguously reached with hnp given an extremely high modal percentage of deviance residuals found outside the simulated envelope (99.9%). The associated mgcViz score of only 24.9% clearly indicated important underfitting for this Poisson GAMM, likely as a result of unmodelled overdispersion (i.e., downwardly biased SE estimates) which prevented to appropriately estimate the uncertainty about the true mean behavior of the process.

A fully‐parameterized NB2 GAMM was then fitted to handle the extra‐Poisson variability and possibly simultaneously the related zero‐inflation (Campbell 2021), which was achieved as confirmed with DHARMa. Assessment checks with hnp and the mgcViz score also confirmed this model's adequacy (Table 1). Then, the implemented “double‐penalty” approach was used to determine whether some of the covariates could be discarded. The resulting reduced NB2 GAMM was then examined to determine if some of its retained smooth terms could rather be converted into parametric components based on their edf values. Then, the final NB2 GAMM was tested for its adequacy and then compared to the initial fully‐parameterized NB2 GAMM to determine whether using fewer parameters provided a more parsimonious fit.

**TABLE 1:** Generalized additive mixed‐effects model (GAMM) specifications and assessments for the three examples presented in this study.

Example	Family^a^	Spline	hnp (%)	*D* ^2^_adj_	mgcViz score (%)
Lake trout	gaussian	Penalized	34.2	91.2^b^	83.6 [81.1, 83.8]
	Gamma	Penalized	34.9	94.0	81.5 [78.4, 83.8]
	**Gamma**	**Penalized**	**26.9**	**94.0**	**81.7 [78.4, 83.8]**
Walleye	nb^c^	Thin‐plate	0.49	25.7	80.1 [75.0, 85.0]
	nb^d^	Thin‐plate	0.71	25.7	80.7 [72.4, 90.0]
	**nb** ^**d**^	**Thin‐plate**	**1.59**	**25.8**	**80.8 [75.0, 87.6]**
Arctic charr	**binomial**	**None**	**0.17**	**54.0**	**92.6 [90.9, 100.0]**

*Note:* The hnp statistic reflects the modal percentage (%) of deviance residuals found outside the simulated envelope based on 10 diagnostic iterations and should be < 10% for a sufficiently adequate GAMM. The adjusted deviance explained (*D* ^2^_adj_) estimates the in‐sample predictive performance. The mgcViz score [95% uncertainty interval] reflects the average percentage of binned deviance residual means within the predicted 80% confidence interval and should have a similar value for a well‐fitting model. Models with regular characters were obtained with maximum likelihood (ML) estimation, whereas final models with restricted ML (REML) estimation are written in bold.

^a^ As referred to in mgcv, such as “nb” for the type‐II negative binomial (NB2) with a log link.

^b^ Corresponds to *R* ^2^_adj_ for a Gaussian GAMM (Zuur et al. 2009).

^c^ Fully‐parameterized model.

^d^ Final model.

#### 2.2.4 Spawning Probability of Female Arctic Charr

In this last example, the spawning probability (π) of anadromous female Arctic charr ( *S. alpinus* ) that were sampled during the upstream migration in three different rivers (*n* = 117) in Nunavik (Québec, Canada) is modeled in relation to their fork length (*FL*, ±1 mm) using data presented in Mainguy, Ouellet, et al. (2025). Given that the occurrence of a female exhibiting developing gonads was a rare event across the three combined sampling locations (13.7%), the estimation of the length‐at‐maturity (*L*_50_), that is, the *FL* at which 50% of the females are expected to exhibit developing gonads, proved impossible on a river‐by‐river basis due to highly unbalanced binary data (see Mainguy et al. 2024 for analytical details). To circumvent this, Mainguy, Ouellet, et al. (2025) previously analyzed the available information from all females and included *river* as a random effect in a binomial GLMM fitted with a complementary log–log link in the glmmTMB package (Brooks et al. 2017). This allowed us to estimate a population‐level *L*_50_ of 574 mm using the fixed‐effect estimates following Mainguy et al. (2024).

Here, *FL*s were binned in 50‐mm classes (hereafter *BIN50*) to analyze them as discrete proportions instead since hnp cannot perform model adequacy assessment for GLMs or GAMs that rely on binary data. This is mostly attributable to the fact that deviance residuals, as for most other types, are difficult to interpret when dealing with a dichotomous outcome (Augustin et al. 2012). A smooth function was first applied to *BIN50*, but this led to edf = 1 and as a result this predictor was refitted as a parametric component. By doing so, the model closely resembled a binomial GLMM, but nonetheless still relied on a penalized quasi‐likelihood maximization to integrate over random effects (Wood 2017), whereas glmmTMB uses instead Laplace approximation (Brooks et al. 2017), such that predictions may slightly differ between the two packages. Following adequacy assessments with hnp and the mgcViz score, the binomial GAMM was also checked for possible overdispersion and zero‐inflation issues that can also arise for the binomial case (Martin et al. 2005; Richards 2008) using DHARMa.

The global *L*_50_ estimate was first calculated from the fixed coefficients of the binomial GLMM‐like GAMM, but because such a frequentist setting only returns estimates ± SE for fixed effects and no measures of uncertainty for variance parameters, a Bayesian setting was independently applied with the R2jags package (Su and Yajima 2024), only in a complementary manner, to provide marginal posterior distributions for all effects. This allowed yielding *L*_50_ estimates with associated 95% credible intervals globally and for each of the three rivers, as detailed in Mainguy, Ouellet, et al. (2025).

## 3 Results

### 3.1 Female Lake Trout

**FIGURE 1:**
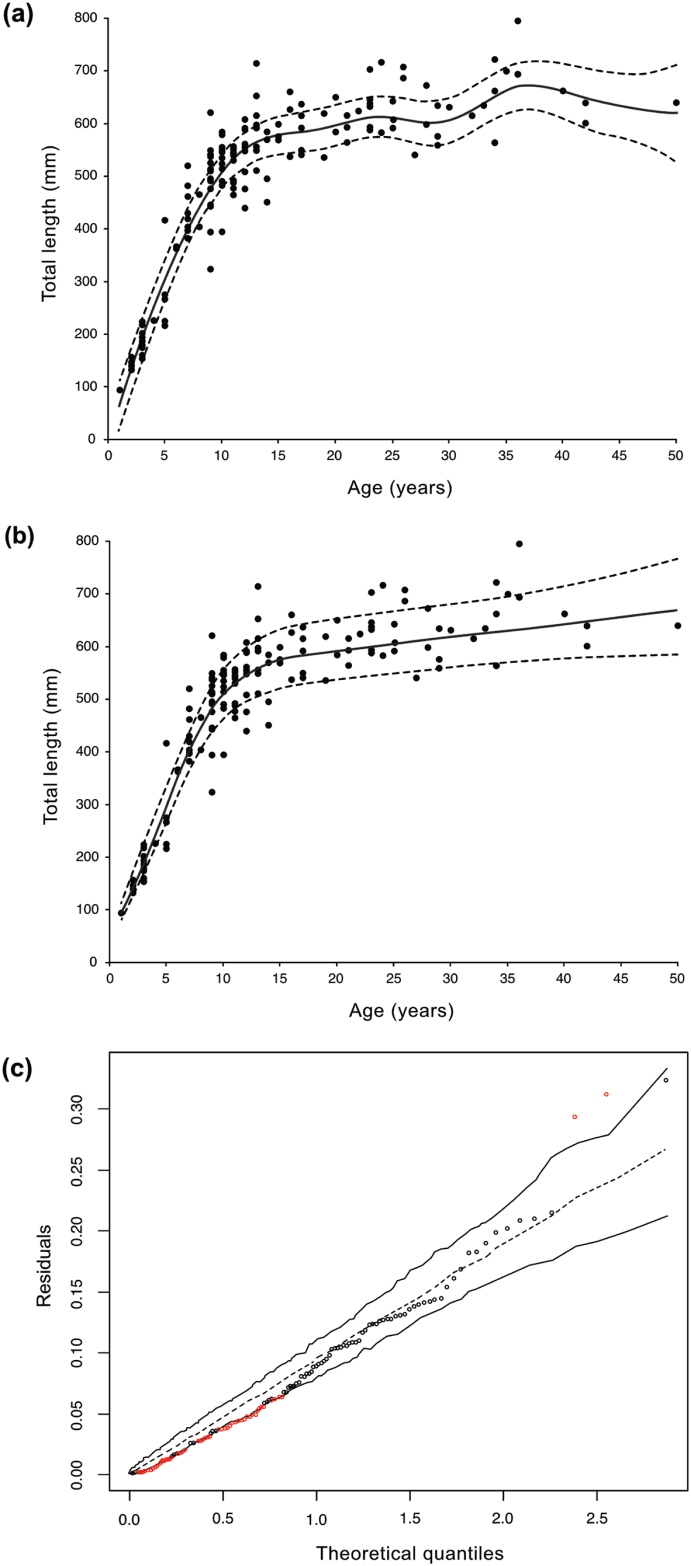
Predicted population‐level length‐at‐age relationships of female lake trout (*n* = 153, filled black circles) sampled from three different lakes. Predictions (with 95% CI) are either based on (a) generalized additive mixed‐effects model (GAMM) fitted with a Gaussian distribution and an identity link using default smooth function settings under maximum likelihood (ML) estimation, or (b) GAMM fitted with the gamma distribution and a log link using an adaptive smooth to further minimize wiggliness under restricted ML (REML). (c) a single half‐normal diagnostic plot with a simulated envelope indicates that the gamma GAMM is inadequate since 36.2% of its deviance residuals are found outside the envelope (shown in red).

The unrealistic predictions of the Gaussian GAMM using default smooth settings are shown in Figure 1a. Using an adaptive smooth instead allowed reaching a monotonic relationship, as shown in Figure 1b for the gamma GAMM. Despite an excellent adjustment of their fitted values to the observed data, both candidate GAMMs were found to be inadequate according to hnp (Table 1). An example of a single hnp diagnostic plot with a simulated envelope for the assessment of the gamma GAMM is presented in Figure 1c. The two inadequate GAMMs were nonetheless judged as still being useful to describe this simple length‐at‐age relationship, with the observed extra‐variation in observed lengths at approximately 5–15 years of age possibly causing such inadequacy due to the variance being either heteroscedastic (Gaussian) or not sufficiently increasing in a proportional manner relative to the mean (gamma) with age.

Comparing these two inadequate candidate models, which both offered at least reasonable approximations of this curvilinear relationship, indicated that the gamma GAMM did not offer a clear improvement in fit over the Gaussian GAMM as it only had a slightly lower ML score (−5.13) for identical Edf as determined from using itsadug. In a consistent manner with the first comparison, however, the Gaussian GAMM was this time clearly preferred by obtaining nearly all the sample‐adjusted Bayesian weight (0.998) compared to its Gaussian counterpart (ΔBIC_c_ = 12.3). The mgcViz score of the preferred gamma GAMM was also slightly closer to the targeted 80% confidence level than that of the Gaussian one (Table 1), altogether suggesting that accounting for the generally increasing variation in length with age provided overall a more accurate fit.

### 3.2 St. Lawrence River Walleyes

Using the “double‐penalty” approach resulted in discarding half of the fixed effects (*n* = 3). Refitting this reduced NB2 GAMM indicated that two of the remaining covariates were now associated to edf = 1 as a result, such that a final NB2 GAMM was refitted with these included as parametric components instead, ultimately leading to excellent adequacy according to hnp with an excellent mgcViz score, but a modest predictive performance (Table 1). Such changes led to a 50% reduction of the Edf associated to the final NB2 GAMM while nonetheless maintaining a nearly identical ML score to that of the fully‐parameterized initial one (*χ* ^2^ = 0.42, df = 8, *p* = 0.999). This more parsimonious, adequate model correctly handled overdispersion ($\hat{\phi }$ = 1.03; *p* = 0.70) and zero‐inflation (ratio of predicted vs. observed zeros of 1.00, *p* = 0.90), and also received 100% of the sample‐adjusted Bayesian weight when compared to its fully‐parameterized counterpart (ΔBIC_c_ = 22.0). It is worth noting that temporal autocorrelation was also likely, such that using itsadug as a possible option to more specifically assess its importance and attempt if needed to adjust for this analytical phenomenon would be the next logical step (see van Rij et al. 2022 for details).

**FIGURE 2:**
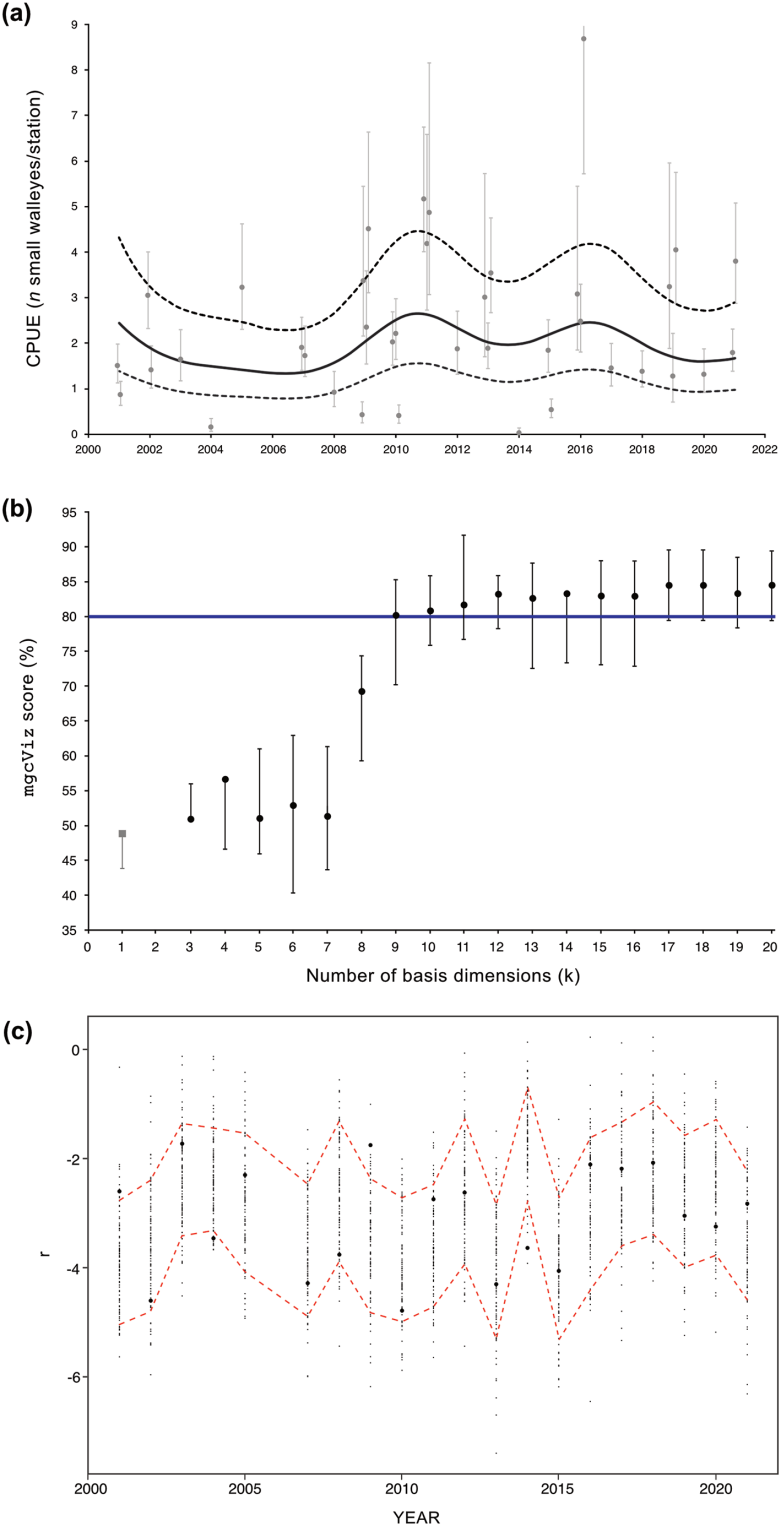
(a) Predicted population‐level temporal variations in the catch‐per‐unit‐effort (CPUE) of small St. Lawrence River walleyes from 2001 to 2021 based on 38 annual surveys that were periodically conducted in eight different interconnected areas. Observed survey‐specific CPUE means (filled gray circles) with associated 95% confidence intervals (error bars) are shown along with the predictions of an NB2 GAMM accounting for multiple sources of variation based on REML estimation (black solid line) with related uncertainty (95% CI, short‐dashed lines). (b) Illustration of the effect of decreasing or increasing the number of basis dimensions (*k*) on the mgcViz score and its 95% “uncertainty interval” (error bars). (c) Example of a single mgcViz diagnostic plot for the preferred NB2 GAMM (*k* = 10), here with a mgcViz score of 80% since 16 out of the 20 binned deviance residual means (filled black circles) are found within the simulated 80% CI limits (red dashed lines).

Lastly, to further illustrate the use of the mgcViz score, we report how fitting *year* as a parametric component or smooth term, with *k* allowed to vary by +1 increments from 3 to 20 (i.e., number of unique years), can possibly lead to under‐ or overfitting. This assessment showed that assuming linearity at the linear predictor scale for *year* resulted in important underfitting, as for smooth terms with *k* ≤ 8 (Figure 2b). Conversely, using *k* ≥ 12 led to slight overfitting that was apparently limited by the in‐built penalization of mgcv, whereas using either *k* = 9, 10, or 11 offered a better realized coverage (Figure 2b). A single mgcViz diagnostic plot for the final NB2 GAMM is shown in Figure 2c to illustrate the underlying principle of the mgcViz score.

### 3.3 Female Arctic Charr

**FIGURE 3:**
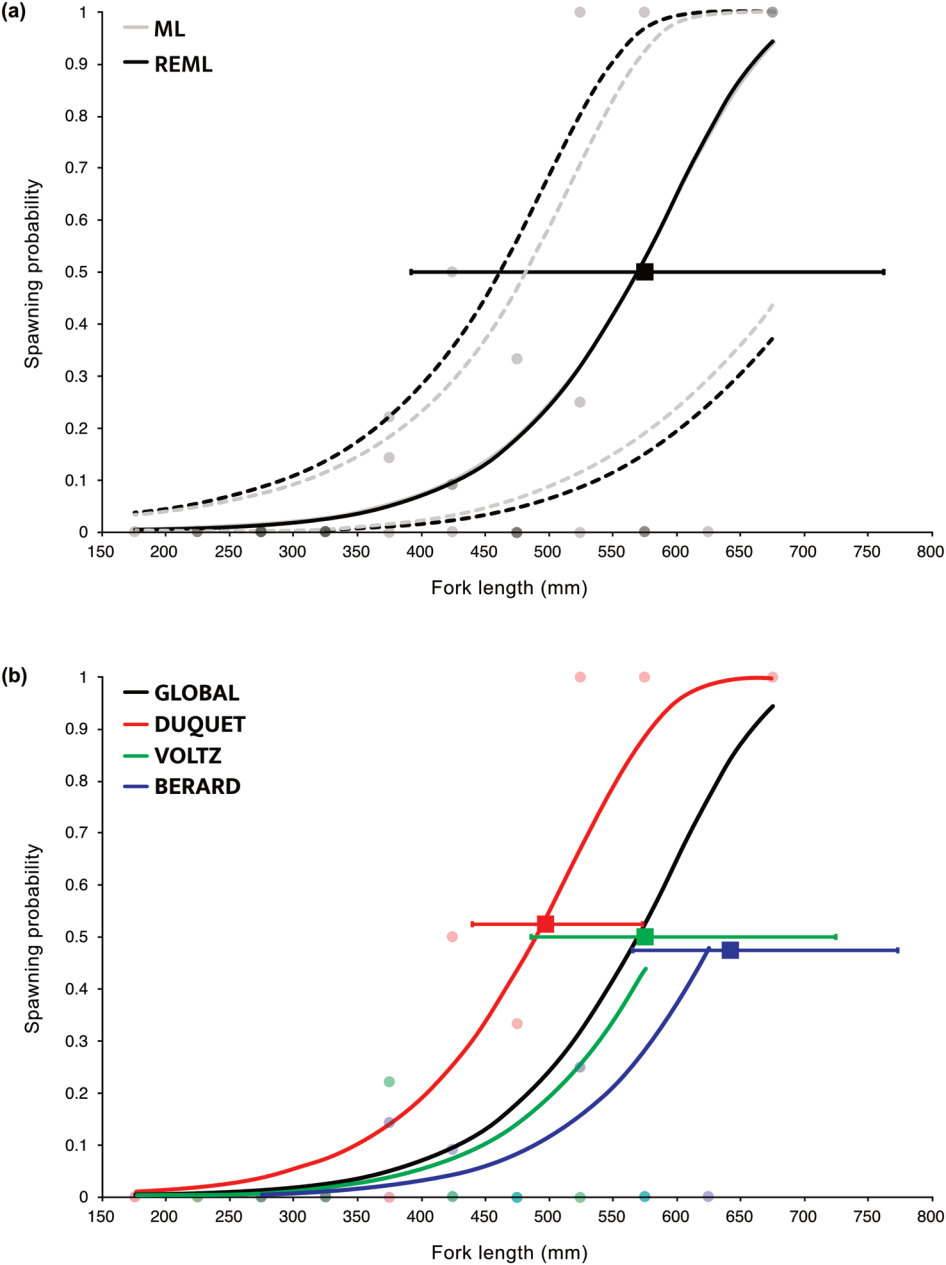
(a) Predicted population‐level spawning probability of female anadromous Arctic charr sampled in three different rivers in relation to their fork lengths when analyzed as discrete proportions using 50‐mm bins (*BIN50*, filled circles) in a binomial generalized additive mixed‐effects model. Predictions obtained under maximum likelihood (ML) and restricted ML (REML) estimation are shown with their respective uncertainties (95% CI, short‐dashed lines). The length‐at‐maturity (*L*_50_; filled black square) as estimated in a Bayesian setting (see text) is also shown with related 95% credible interval (error bar). (b) Predicted *river*‐specific reproduction ogives from the binomial GAMM presented in (a), with related color‐coded observed discrete proportions (shadowed filled circles). The *river*‐specific *L*_50_ estimates (filled squares) are jittered to better visualize their 95% credible intervals (error bars).

The binomial GLMM‐like GAMM was sufficiently adequate according to hnp, offered reasonably good predictive performance, but overfitted the observed discrete proportions (Table 1). The discrete proportions being modeled were sufficiently equidispersed ($\hat{\phi }$ = 1.33; *p* = 0.27) and although the binomial GAMM only predicted 91.8% of the observed zeros, this was tolerable from a statistical standpoint (*p* = 0.71). The resulting global ogive was however associated with important predicted uncertainty under ML and even more so under REML estimation (Figure 3a). For sake of clarity, only the central tendencies are shown for the predicted *river*‐specific ogives obtained under REML estimation (Figure 3b). The global *L*_50_ was estimated at 569 mm under the frequentist approach, whereas the use of a Bayesian setting provided an estimate of 575 mm [392, 763] (Figure 3a). Despite slight mismatches between the *L*_50_ estimates obtained from a Bayesian setting and the corresponding predicted *FL* intersecting at $\hat{\pi }$ = 0.5 for the *river*‐specific ogives, a similar interpretation of the reproduction of anadromous female Arctic charr was reached as in Mainguy, Ouellet, et al. (2025). The important related predicted uncertainty, likely generated by the low sample sizes (*n* = 26) resulting from the binning the females in 50‐mm *FL* classes (i.e., *BIN50*), is thought to have mainly caused the detected overfitting as captured by the mgcViz score.

## 4 Discussion

As for GLMs fitted with commonly‐used R packages such as stats and glmmTMB (e.g., Mainguy and Moral 2021), GAMs and their mixed‐effects counterparts fitted with mgcv can now also be assessed for their adequacy using hnp. Assessing for possible under‐ or overfitting with the proposed mgcViz score also makes it possible to evaluate if the signal in the data is apparently correctly captured by the predicted relationship of a GAM for a given predictor of interest. These additions, when jointly employed with tools implemented in available diagnostic‐oriented packages such as DHARMa, may altogether offer a more thorough assessment regarding the adequacy of GAMs.

A noteworthy advantage of mgcv that we want to highlight here is that it can also fit GLMs by simply not applying a smooth function to the considered predictor(s), as in the last example with female Arctic charr. As a result, candidate GLMs and GAMs attempting to describe the same ecological data can be assessed and compared within the same modeling framework (Nelsen et al. 2023; Mainguy, Paradis, and Moral 2025) and, more importantly, with the same diagnostic tools such as those proposed in this study. The use of ML estimation when fitting GLMs in mgcv also conveniently provides nearly (if not) identical parameter estimates and related statistics to those obtained in GLM‐oriented packages, such as glmmTMB, for shared distributions, especially so when only fixed effects are used. Moreover, since the linearity assumption of GLMs is challenging to assess (Heit et al. 2024), the use of smooth functions within mgcv also allows us to easily examine whether linearity at the linear predictor scale is more probable by checking whether a smooth term has edf = 1, thus offering a rapid assessment for such purpose.

Lastly, reporting *R* ^2^_adj_ for Gaussian GAMs, as directly provided by mgcv, or *D* ^2^_adj_ for non‐Gaussian GAMs (as in Table 1), is advisable given that it is also part of the evaluation about the usefulness of a (ideally) correctly‐specified “best” model (Mac Nally et al. 2018). For instance, a given GAMM may be sufficiently adequate to statistically describe an ecological relationship, but nonetheless be associated with a modest *D* ^2^_adj_, as in the second example about the St. Lawrence River walleyes. In such a case, the “best” adequate model offers a rather limited ability to explain the observed variation in the response variable and such statistical reality therefore needs to also be acknowledged. As noted by Low‐Décarie et al. (2014), statistical models have progressively become more complex over time, such as the use of GAMMs that incorporate many smooth terms, but their related predictive performance has oppositely been gradually less reported. Altogether, we believe that reporting *R* ^2^_adj_ and *D* ^2^_adj_ when possible, together with other performance metrics (e.g., Becker et al. 2020), should ideally be sought at all times.

Overall, the three MELCCFP ecological examples presented in this study provided statistical support for the addition of nonlinearities in the modeled relationships for the first two cases, whereas the last one instead showed that using mgcv and its gam() function can closely mimic a GLMM to produce a classical binomial (sigmoidal) regression curve. In all instances, the GAMMs fitted with different distributional assumptions were appropriately assessed for their adequacy with hnp, whereas suspected overfitting could be detected in the last example from using the mgcViz score, although for other reasons than allowing too much flexibility for a smooth term. The effect of not allowing for sufficient flexibility by voluntarily reducing *k* in the second example also demonstrated that this could sometimes lead to important underfitting. Interestingly, these examples illustrated quite diverse analytical situations where the adequacy, possible under‐ and overfitting, and in‐sample predictive performance could sometimes point in quite different directions. Although these GAMMs were all ultimately considered useful to statistically describe their corresponding nonlinear ecological relationship, the adequacy tools presented in this study, on top of *D* ^2^_adj_, further helped to interpret the ecological significance of their results from a more comprehensive statistical standpoint.

## Author Contributions

**Julien Mainguy:** conceptualization (lead), data curation (lead), formal analysis (equal), investigation (lead), methodology (lead), project administration (lead), software (equal), supervision (lead), validation (equal), visualization (lead), writing – original draft (lead), writing – review and editing (equal). **Rachel McInerney:** formal analysis (equal), investigation (equal), methodology (equal), validation (equal), writing – review and editing (equal). **Russell B. Millar:** conceptualization (equal), formal analysis (equal), methodology (equal), writing – review and editing (equal). **Eliane Valiquette:** writing – review and editing (equal). **Martin Bélanger:** writing – review and editing (equal). **Rafael de Andrade Moral:** conceptualization (equal), formal analysis (equal), investigation (equal), methodology (equal), software (lead), writing – review and editing (equal).

## Funding

The data used in this study were all acquired by the Ministère de l'Environnement, de la Lutte contre les changements climatiques, de la Faune et des Parcs, which funds and monitors its fish and wildlife projects.

## Conflicts of Interest

The authors declare no conflicts of interest.

## Data Availability

The data and R scripts that support the findings of this study are openly available in GitHub at https://github.com/julienmainguy/GAM‐adequacy.
